# Clinical Notes from the Service of Prof. Péan, Hôpital St. Louis, Paris

**Published:** 1879-01

**Authors:** W. S. Caldwell

**Affiliations:** Paris


### ARTICLE XI.

### CLINICAL NOTES FROM THE SERVICE OF PROF. PÉAN, HÔPITAL SAINT LOUIS, PARIS.

Though Prof. Péan is not a very dashing or brilliant operator as far as the mechanical execution of his surgery is concerned, yet as a teacher he is undoubtedly one of the first, if not the very best in Paris.

The Saint Louis, which contains 853 beds, is situated in the midst of a great manufacturing district of the French capital, and is devoted mostly to surgical cases.

This great amount of material is systematically arranged and presented for the purpose of teaching. Cases are grouped together in such a manner that we are generally able to see several patients affected with the same disease during a single day, as well as the operations done on them.

Tumors and other morbid growths are studied first in their general aspect, and then microscopical specimens of the same are prepared which all have a chance to carefully examine.

On Saturday, Nov. 2d, the first cases brought into the amphitheatre were two women suffering from uterine fibroid.

The Professor said: "I bring before you to-day two cases of disease among the most interesting known to the domain of either medicine or surgery; interesting because we are sometimes able to do wonders in the way of curing our patient, and at others are absolutely helpless to render her the least assistance.

"The first thing to be done when a case of uterine fibroid presents itself to you for treatment, is to ascertain by a careful examination the exact location of this growth in its relation to the uterus itself. In other words, Is the tumor extra-uterine, intra-mural or intra-uterine? The first of these cases that will occupy our attention is a woman 36 years of age, who has borne one child, now fifteen years old. The tumor, in her case, though occupying the interior of the womb, is attached and bound down to the walls of the uterus in its whole extent by the mucous lining of that organ, and is therefore termed a sub-mucous fibroid. It is as large as a child's head one year old, and occupies one side and the posterior aspect of the uterine cavity.

"When the patient presented herself to me three weeks ago, she had been flooding for many months, and was consequently very anæmic.

"Although in the management of these cases there is no single line of treatment that has not its advocates and its opponents, yet all, I believe, are agreed that a thorough dilatation of the mouth of the womb exercises generally a beneficial influence at least upon the hæmorrhage that is so often one of its most dangerous and perplexing symptoms. A great many devices have been used to effect this object.

"The one that I prefer, and the one which I practiced on this patient three weeks ago, was to incise the mouth of the womb freely with Dr. Paquelin's thermo-cautery. I first make my incisions laterally, and if they do not open the parts sufficiently freely I also incise the anterior and posterior lips.

"The advantages of this procedure over the knife or the scissors are that you have no hæmorrhage, and also that your cut surfaces do not so readily heal as they do when made by a cutting instrument.

"After I operated in this manner on this woman, she nearly ceased flooding, and has lost but little blood since.

"I shall now proceed to perform the next step in the operation in this case, as the womb is now well open, and I can get free access to the tumor. This second step in the management of our case consists in puncturing this mass at several points with the conical point of the thermo-cautery."

He then produced anæsthesia with chloroform, introduced a large wooden cylindrical speculum, after which he punctured the fibroid mass at three points with his thermo-cautery, introducing it to a distance of about six centimeters. He then proceeded to incise the mouth of the womb in the second case as he had done in the first, and proposes to treat her, after a few weeks, in the same manner as he did the first case described.

I saw the first of these patients twice after she was operated on. She had a good deal of discharge from the uterus, though an insignificant amount of constitutional disturbance. At the end of two weeks she was sent out of the hospital, to remain for a month, at the end of which time she will return and have the operation of puncturing this tumor repeated.

Prof. P. claims that in several cases this mode of treatment has been very successful in his hands, the reaction produced by the introduction of the cautery leading to the absorption of the morbid growth.

Carl Braun, of Vienna, would manage a case like this by first dilating the mouth of the womb well with a sponge tent and then making a free incision through the mucous covering of the tumor from top to bottom, then giving ergot freely to bring on uterine contractions.

Prof. Shröder, of Berlin, is opposed to meddling with these cases in any way if they are very anæmic until improvement in the general condition of health has been obtained. He has, he says, so often seen the most disastrous results follow the least disturbance of these cases where the patient is very bloodless, that he dreads them more than almost any class of cases that come under his observation.

In a case that I saw him manage he dilated the womb and then used cold injections of a solution of carbolic acid and proposed to wait before doing anything more active until his patient was in a better physical condition. There is no principle in surgery now more generally recognized than that which associates as cause and effect the septic processes that follow operations and an extremely anæmic condition of the subject operated on.

Saturday, Nov. 9th, 1878. Prof. Péan brought into the amphitheatre to-day a number of cases of bursal tumors situated at or near the wrist joint. He said: "A great variety of plans had been adopted in the treatment of these cases; but the first step to be taken was to determine with *accurracy* whether the bursa communicates by a fistulous opening with the joint *itself*, or whether it simply involves the tendon of the muscle.

" If you find that by firm pressure you are able to press the contents of the bursa out into the joint, then great care must be observed in its management. This last condition of things, however, is the great exception.

" The failure to make this simple distinction in these tumors has led to an endless amount of useless discussion as to the best manner of treating them."

He opens them by a large valvular incision, keeping the knife in the wound until all the fluid is pressed out. He then uses pressure on the seat of the tumor by means of a rubber band encircling the wrist, a short wide piece of wooden splint being put under the band, to keep it from exercising too much pressure upon the circulation of the hand.

Prof. Lister opens these bursæ under the spray and dresses them antiseptically without the least fear of any evil results.

In obstinate cases of "housemaid's knee," I saw Prof. L. open the bursa in the same manner, keeping the patient in bed for a few days, and they recovered without any inflammatory or constitutional disturbance following the operation.

Saturday, Nov. 16th, 1878. The first case shown us to-day was that of a girl 18 years of age and apparently in good health. Prof. Péan said : " The first case I show you to-day is one of great interest in a diagnostic point of view. The tumor that I show you occupying the axillary region in this young girl, is about the size of a goose-egg and was first observed about nine months ago.

" It grew very slowly until the last three months, when it began to increase in size rapidly, and has lately, by its pressure upon the axillary plexus of nerves, caused the patient a great amount of suffering. The first question that we ask ourselves is, What is this growth ? What are we most likely to have in this region ?

" Adenitis involving the lymphatics, will be the very natural answer. This adenitis may be acute or chronic, it may be suppurative in character, or it may be accompanied by a simple hypertrophy of the gland-structure with no tendency to a breaking down of its substance.

"But the case we have before us differs essentially in its physical aspect from either of these usual forms of glandular disease found in this region. First, we have no fluctuation, and have not therefore to deal with a cyst. Second, when I take hold of this tumor and attempt to move it, I find it firmly attached to the pectoral muscle that underlies it. I find, in fact that the growth has invaded the tissues in every direction. The skin is not yet involved, on account, perhaps, of the great thickness of adipose tissue that lies beneath it. I have not therefore the slightest hesitation in pronouncing this growth one of a malignant type."

He then cut down upon the tumor by a long incision, extending from the middle of the axilliary space down the outer aspect of the mammary gland a distance of about 18 Cm. He then began below, and dissected up the mass from the pectoral muscle, and when he had nearly reached the upper border of the tumor, he remarked that as he found it attached pretty firmly to the sheathes of the large plexus of vessels in this region, he must proceed with great caution. Cutting at the same time nearer the growth with his knife, he cut into it, and a large quantity of healthy pus flowed out and the whole thing collapsed. It was simply an indolent form of suppuration of one of the lymphatic glands. Its sac had taken on an inflammatory change and had involved the surrounding tissues in the same process.

Prof. P. at once recognized and acknowledged his error in diagnosis, but proceeded to carefully dissect out the sac, and consoled himself by remarking that his operative procedure was probably the best manner of treating this cyst, for if he had simply opened it and injected its cavity with tincture of iodine he might have had great difficulty in curing it, whereas by this single operation recovery was sure to follow.

Among other cases of interest operated on at this date was a young man, aged 19 years, who, eighteen months before, was taken with an indolent form of orchitis, which gradually went on to suppuration, and in spite of all treatment the process had continued up to this time. Lately the patient's health had begun to fail, and Prof. Péan believed that the testicle was affected by a tuberculous form of disease, and that the patient was in imminent danger of having his whole system contaminated by this tubercular poison.

He proposed castration as the only remedy. He dwelt at length on the necessity of treating this class of cases promptly and intelligently, and said that, in the early part of his professional life he had allowed many cases of this kind to run into a general tuberculosis of the lungs by delaying the operation that he now proposed to perform on this patient.

### OCCLUSION OF THE VAGINA.

Saturday, Nov. 23, 1878.—Our first case to-day was that of a woman 32 years of age, suffering from occlusion of the vagina. Three years ago she had had her first child. Following this she had some form of uterine disease, for which she was treated by a physician in one of the provincial towns. About eight months ago she had a very strong application made to the mouth of the womb, followed by a good deal of pain and vaginal discharge.

Since that time she had not menstruated, and had lately sufferred a good deal from pain in the lower part of her abdomen. Examination by palpation showed her womb considerably enlarged, extending eight or ten centimeters above the symphysis pubis. The finger could only be introduced into the vagina for a distance of two and one-half centimeters, at which point it came in contact with a firm resistance which could not be overcome.

He said that occlusion of the vagina was not a very infrequent accident following the use of very strong caustic applications to the mouth of the womb. He had no doubt but that the acid nitrate of mercury had been used in this case, but even the free cauterization of the mouth of the womb by the nitrate of silver might be followed by this untoward result if care were not taken to free the upper part of the vagina from all excess of the substance used. If this occlusion were only a kind of band or bridle, thin in texture, we might break it up with the finger.

After attempting this mode of procedure and failing in it, he introduced a large sound into the bladder and the forefinger of the left hand into the rectum, as guides to the direction in which he was to make his incision, and using a long bistoury he gradually cut his way through the occluded part of the vaginal walls. A considerable amount of a black, tarry-looking fluid flowed out when he had passed through the occlusion. The menstrual fluid which had been dammed up by the obstacle that prevented its escape had burrowed down somewhat in the direction of the vaginal outlet, and so had considerably facilitated the operation of reaching the mouth of the womb.

He proposes to keep the parts open by the introduction twice a day of a quadrivalvular speculum, gradually extending the blades of the same until the vaginal cavity is of the requisite size. He considers this manner of dilating and keeping the parts open as better than the use of any of the special dilators made for that purpose.

I treated myself, a few years ago, a poor woman who very nearly suffered from this same accident, by the attempted application of a chloride of zinc paste to the mouth of the womb, for the removal of what was *supposed* to be a cancerous tumor.

The person who used this "cancer plaster" did not get his application to the mouth of the womb but applied it to one side of the cervix, the effect of which was to destroy a considerable portion of the left wall of the vagina at its junction with the neck of the womb itself. When the wound healed the contraction of the cicatrix that remained drew the upper walls of the vagina together, narrowing this portion of its cavity to about one-third of its natural capacity.

The cervix uteri was also drawn considerably towards the left side, the body of the same falling towards the right, thus producing a latero-version of that organ. The result of this alteration in the texture and relation of the parts was to render coitus very painful, produce a bearing down sensation when the patient was on her feet, and in fact to render her a perfect invalid. For the relief of these symptoms I attempted to divide these cicatricial bands first by a number of superficial incisions with the knife. Fear of hæmorrhage deterred me from making my incisions very deep so I made but little progress in the treatment of my case until it occurred to me to try the elastic ligature. I threaded a large curved needle with one of these ligatures, passed it deeply into the parts and tied it firmly. At the end of twenty-four hours it had cut its way through the hard mass and I had the satisfaction of having an incision deeper than I would have dared to have made with the knife. By repeating this process at other points in this band I was able to partially overcome the deformity and considerably benefit my patient.

In the last students' number of *Le Progrès Médical* there are several pages devoted to the system of medical education in vogue in the different countries of Europe as well as a review of the medical school system in the United States.

The writer says that *unfortunately* the State does not control medical education in America. That our students begin often with no primary education, have generally greatly inferior advantages for the pursuit of their studies, and secure degrees after studying one-half of the time that is required in France to get a permission to practice as a veterinary surgeon.

Unfortunately and unjustly our whole profession are suffering in the eyes of foreign physicians for the short-comings of our medical educational system.

In Germany I found intelligent professors who had an impression that all the medical schools of Philadelphia sell their diplomas after the fashion of the eclectic concern that has been not only a disgrace to our profession but to the good name of every American.

W. S. CALDWELL.

PARIS, Nov. 30th, 1878.

---

LIEBIG'S EXTRACT OF MEAT.—When Baron Liebig stated that his discovery of Extract of Meat would prove one of the most beneficial to mankind of his various discoveries, he had no conception of the enormous extent of consumption which the future would develop. From a few hundred Bavarian oxen, which were originally sufficient to supply the continued demand, the total of cattle since used by the Liebig Company, exceed 1,500,000 head.

